# Optimization of Coupling Efficiency in Butterfly Optical Communication Laser Based on Chaotic Adaptive Seeker Optimization Algorithm

**DOI:** 10.3390/mi14071417

**Published:** 2023-07-14

**Authors:** Shunshun Zhong, Cong Xu, Dongmei Sun, Lian Duan, Ji-an Duan

**Affiliations:** 1 State Key Laboratory of Precision Manufacturing for Extreme Service Performance, College of Mechanical and Electrical Engineering, Central South University, Changsha 410083, China; 2 Changsha Aeronautical Vocational and Technical College, Changsha 410124, China

**Keywords:** coupling efficiency, chaotic disturbance, seeker optimization algorithm, multi-degree of freedom alignment

## Abstract

A chaotic adaptive seeker optimization algorithm (CASOA) is proposed in this study to improve the coupling efficiency and accuracy of a butterfly optical communication laser. It primarily relies on chaotic disturbance to improve seeker search performance. The chaotic disturbance enables the algorithm to jump out from local extremes. Furthermore, chaos is associated with a novel strategy for optimizing search paths with a small population. A simulation and experiment are conducted to demonstrate that the CASOA with a few seekers has an excellent search success rate with few iterations in the coupling alignment. These results indicate that the proposed CASOA can reliably improve the coupling accuracy and efficiency of laser diodes and single-mode fibers.

## 1. Introduction

Butterfly-encapsulating communication lasers have recently been widely adopted in the tracking system and electronic industries [1]. The coupling efficiency of the laser diode (LD) and the fiber determines the final output power of the laser and thus has an impact on optical transmission performance [2,3]. In general, the coupling process comprises three platforms (the X, Y, and Z axes) with three degrees of freedom when moving to a certain location. A laser diode is clamped on one of the platforms, and a fiber is clamped on the other. The laser can output the highest optical energy after moving and aligning stages in three directions. However, the optical energy of the initial position is low due to the different centers of the mode field and assembling errors. The relative positions are then constantly adjusted using advanced algorithms between the LD and fiber until the target power value is reached. Consequently, a reliable algorithm with high precision and efficiency is desirable for optical device coupling.

Several coupling methods have been investigated to date [4,5,6,7,8]. Because of the traps in the local optimum, some traditional group optimization algorithms have a low success rate and are unsuitable for fast coupling. Lian et al. [9] recently proposed a particle swarm optimization (PSO) algorithm with a decreasing law of inertia in a coupling alignment between an LD and a single-mode fiber (SMF). Their experimental results demonstrated an exceptional coupling efficiency and success rate. The critical point of their strategy was the early improvement of the global search. However, because the PSO algorithm relied on many particles to exchange optimal information for the execution of the next iteration, it was unsuitable for quickly searching optical signals. When the number of particles decreased, so did the success rate of searching [3,9]. Consequently, we attempted to improve the coupling efficiency using a group optimization algorithm with few particles.

The seeker optimization algorithm (SOA) achieves the global optimum in a robust and fast manner [10,11,12,13]. Unlike the PSO, the SOA can narrow the neighborhood search range when searchers are in better positions and can expand the search range when searchers are in inferior positions. The SOA can find the optimal solution for numerical and qualitative problems due to its complex human understanding and dynamic adjustment. Meanwhile, premature convergence limits the applications in complex optimal problems (especially those with a large number of local optima). Yang [14] promoted the adaptive adjustment capability in the initial iteration stage of the SOA through a dynamic adaptive search step (DASSS-SOA). This algorithm has been combined with a wavelet neural network to successfully predict network traffic. However, during the iteration process, this algorithm cannot escape the local optima. Furthermore, few studies on the SOA have been conducted in a small number of particles. The chaos uses ergodic characteristics to adjust the search sequence throughout the search process. Several studies [15,16,17] have used the chaotic method to optimize the scanning space in various swarm optimization algorithms. We proposed a novel SOA-based optimization strategy in this study to solve the coupling efficiency and robustness of the LD and SMF in butterfly communication lasers. The chaotic method and adaptive step length adjustments were integrated into the SOA (CASOA). The proposed CASOA, dynamic adaptive search step size SOA (DASSS-SOA [14]), linear SOA (LSOA [18]), and linear PSO (LPSO) [9] were simulated and tested to compare the search performance of several algorithms in a small number of particles (seekers). Finally, the results were discussed, and the benefits and limitations of the above algorithms were evaluated.

## 2. Optimization of the Seeker Optimization Algorithm

### 2.1. Seeker Optimization Algorithm

The SOA is a swarm intelligence optimization algorithm based on human cooperative behavior [10,11,12,13,14,15]. The SOA considers the team as the search population and the position of the searchers as the candidate solution to the optimization problem. Then, it determines the search direction by simulating behaviors, such as egoism, altruism, and proactivity. To complete the coordinate iteration, it determines the step length by simulating the mental characteristics of human search [10]. The mathematical models expressed in Equations (1)–(3) are as follows:

(1) $${\vec{d}}_{i,ego}\left(t\right)=sign\left({\vec{p}}_{i,best}-{\vec{x}}_{i}\left(t\right)\right)$$

(2) $${\vec{d}}_{i,alt}\left(t\right)=sign\left({\vec{g}}_{i,best}-{\vec{x}}_{i}\left(t\right)\right)$$

(3) $${\vec{d}}_{i,pro}\left(t\right)=\left\{\begin{array}{c} {\vec{d}}_{i}\left(t-1\right),\;f\left({\vec{x}}_{i}\left(t\right)\right)>f\left({\vec{x}}_{i}\left(t-1\right)\right)\\ -{\vec{d}}_{i}\left(t-1\right),\;f\left({\vec{x}}_{i}\left(t\right)\right)\leq f\left({\vec{x}}_{i}\left(t-1\right)\right) \end{array}\right.$$

where ${\vec{d}}_{i,ego}$ denotes the egoistic direction, ${\vec{d}}_{i,alt}$ is the altruistic direction, and ${\vec{d}}_{i,pro}$ is the proactiveness direction which is searched by the *i*-th seeker when the iterative algebra is t. ${\vec{g}}_{i,best}$ implies that the entire population of seekers has found the optimal position, while ${\vec{p}}_{i,best}$ implies that the i-th seeker has found the optimal position. Thus, $f\left({\vec{x}}_{i}\left(t\right)\right)$ is the fitness value which is searched by the *i*-th seeker when the iterative algebra is t. Similarly, $f\left({\vec{x}}_{i}\left(t-1\right)\right)$ is the fitness value when the iterative algebra is t−1. The stochastic weight averaging method [19] is used to determine the final search direction considering the above three directions. The expressions are as follows:

(4) $${\vec{d}}_{i}\left(t\right)=sign\left(\omega_{0}{\vec{d}}_{pro}+\varphi_{1}{\vec{d}}_{ego}+\varphi_{2}{\vec{d}}_{alt}\right)$$

(5) $$\omega_{0}=\omega_{0max}-\frac{t\left(\omega_{0max}-\omega_{0min}\right)}{t_{max}}$$

where $\varphi_{1}$ and $\varphi_{2}$ are random numbers with mutual independence that follow the $\left[0,\;1\right]$ distribution; $\omega_{0}$ is the inertia weight; $sign\left(.\right)$ is the symbolic function; and $\omega_{0max}$ and $\omega_{0min}$ represent the maximum and minimum of the inertia *weight*, respectively.

The membership function of the step length can be expressed by the Gaussian function because the property of the Gaussian function is consistent with the nature of the human mind. The corresponding expression is

(6) $$u\left(\alpha \right)=e^{-\frac{\alpha^{2}}{2\delta^{2}}}$$

Herein, u is the degree of membership, and its value is below 0.0111 when the step lengths α, which are the fuzzy reasoning inputs, are located outside $\left[-3\delta ,3\delta \right]$. Thus, according to the property of the Gaussian function, the minimal step lengths of α find the minimal u, which is 0.0111. The degree of membership depends linearly on the fitness of the individual seeker and can be expressed as

(7) $$u_{i}=u_{max}-\frac{s-I_{i}}{s-1}\left(u_{max}-u_{min}\right)$$

where $u_{max}$ is the maximum degree of membership, which is equal to or close to one. Furthermore, $u_{min}$ is the minimum degree, set to 0.0111. Then, s is the degree of the whole population, and $I_{i}$ is a serial number of the *i*-th seeker after sorting the fitness in ascending order. To develop the randomness of each dimension and the local search ability of the algorithm, the step length $\alpha_{ij}$ is

(8) $$\alpha_{ij}=\tau_{i}\sqrt{-\ln\left(u_{ij}\right)}\;$$

where $\alpha_{ij}$ is the step length in the next movement, $u_{ij}$ is the step length membership which is searched by the *i*-th seeker in the *j*-th dimension and is directed to distribute randomly, and $\tau_{i}$ is the Gaussian membership function parameter and can be expressed as

(9) $${\vec{\tau }}_{i}=w_{1}\cdot abs\left({\vec{g}}_{best}-{\vec{x}}_{rand}\right)$$

(10) $$w_{1}=\left(t_{max}-t\right)/t_{max}$$

where ${\vec{g}}_{best}$ is the location of the optimal seeker in the current population and where ${\vec{x}}_{rand}$ is a random location in the search coverage of the current population. Then, w denotes the weight that changes with the number of iterations to improve the search accuracy during the later search stage, and $t_{max}$ represents the maximum iterative algebra. To solve the uniform degree of membership, we connected the degree with the fitness of the individual seeker. After obtaining the step length and direction, the updated position of the seekers is

(11) $$x_{ij}\left(t+1\right)=x_{ij}\left(t\right)+\alpha_{ij}\left(t\right)d_{ij}\left(t\right)$$

**Figure 1:**
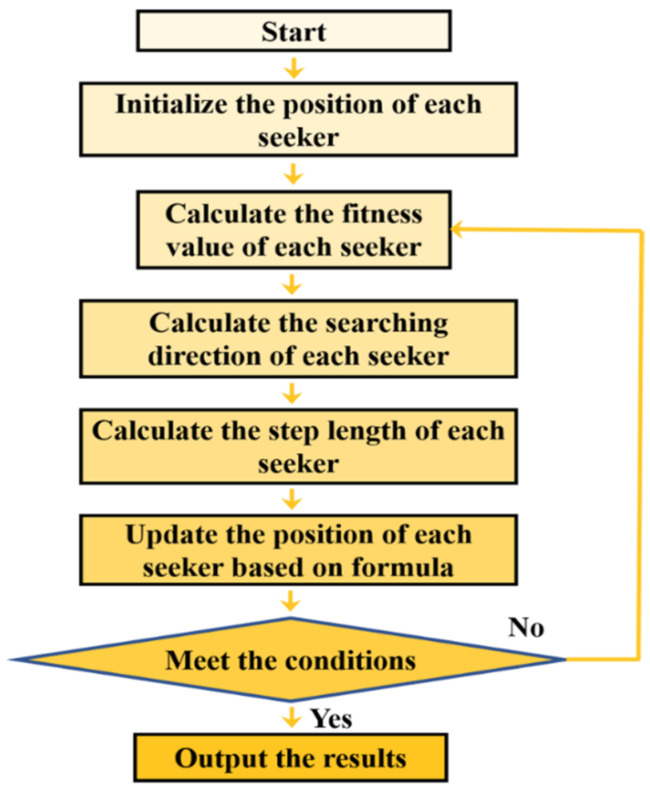
Flowchart of the linear seeker optimization algorithm.

The flowchart of the linear SOA is shown in Figure 1.

### 2.2. Optimization Strategy

In the linear SOA [10], the step length $\alpha_{ij}$ critically affects the search performance. Equations (9) and (10) demonstrate that the inertia weight $w_{1}$ can adjust the search step length gradually via the linear decreasing law. However, in industrial applications, some complex problems cannot guarantee that a newly arrived position achieves a higher fitness value at an early stage than before. If the fitness value reaches the local optimum and the search step length is reduced while the inertia weight decreases continuously, the search will stagnate and end. Furthermore, determining the maximum iteration is challenging and impacts the algorithm adjustments. To improve the inertia weight in Equation (10), the DASSS-SOA adopted an adaptive adjustment law [15]. In nonlinear law, the inertia weight changes dynamically, causing an automatic change in the step length. The search position $x_{ij}(t)$ is then moved to the next iteration $x_{ij}(t+1)$ as the step length varies. However, the proposed DASSS-SOA lacks the ability to jump out from the local optimum during the search process. In this study, the chaotic map is introduced to initialize the seekers, and the chaotic disturbance is introduced to help seekers escape from their current locations to improve the convergence performance of the SOA.

**Figure 2:**
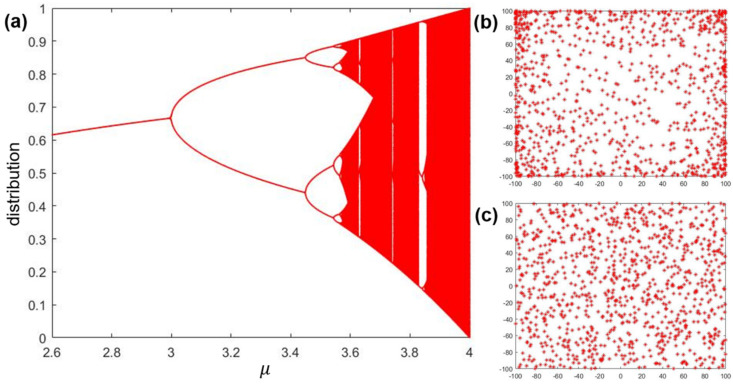
(**a**) The trajectory of the logistic map among the range 2.6–4; (**b**) the distribution of 1000 particles followed the logistic map; and (**c**) the distribution of 1000 particles was random.

The chaotic initialization of seekers is essential for SOA distribution because it allows better positions to replace worse positions. The logistic map is a common chaotic model, and the one-dimensional mapping expression is [20,21]

(12) $$\xi_{n+1}=\mu \xi_{n}\left(1-\xi_{n}\right),\;{\;\xi }_{n}\in \left[0,\;1\right]$$

where μ is a variate that controls the generation of the logistic chaotic sequence (range: 2.6–4). The trajectory of the logistic map is shown in Figure 2a. As demonstrated in Figure 2a, when μ=4, the Lyapunov index [22] is 0.69, and the generated points are widely distributed in the interval [0, 1]. Thus, we set μ=4 to establish chaotic initialization in the SOA. The initialization steps are described as follows:

Step #1: This step sets up the upper (*U_b_*) and lower (*L_b_*) limits of the search space, the maximum number of iterations *t_max_*, and the number of seekers *n*.
Step #2: This step generates a vector $X_{i}^{d}$ with a dimension *d* at random in the search space [0, 1] ($X_{i}^{d}\neq \;0,\;0.25,\;0.5,\;0.75,\;1)$ and then derives *n* vectors via the logistic map (Equation (12)).
Step #3: This step carries all the vectors to their corresponding range of initial positions, with the following relationship:
  (13) $$\xi_{n+1}^{\prime }=L_{b}+\left({U_{b}-L}_{b}\right)\xi_{n+1}$$

Figure 2b,c show a comparison of logistic and random initializations. The number of vectors in the test is 1000, and the spatial boundary is ±100. It is clear that the points in Figure 2b tend to the space boundary. The findings show that the logistic map facilitates the extent of exploration. Furthermore, based on the logistic map, the particles achieve better positions for the next iteration after redistribution.

After initializing the position of the seekers, the SOA enters into an iterative process with chaotic disturbance. The chaotic disturbance alters the position equations of the seekers in various ways (shown in Equations (14)–(16)).

(14) $$\left\{\begin{array}{c} {{\vec{d}}_{i,alt}}^{\prime }\left(t\right)=sign\left({\vec{g}}_{i,best}^{\prime }-{\vec{x}}_{i}\left(t\right)\right)\\ {\vec{g}}_{i,best}^{\prime }={\vec{g}}_{i,best}+∆ \end{array}\right.$$

(15) $$\left\{\begin{array}{c} {{\vec{\tau }}_{i}}^{\prime }=w_{1}\cdot abs\left({\vec{g}}_{i,best}^{\prime }-{\vec{x}}_{rand}\right)\\ {\vec{g}}_{i,best}^{\prime }={\vec{g}}_{i,best}+∆ \end{array}\right.$$

(16) $${x_{ij}}^{\prime }\left(t+1\right)=x_{ij}\left(t\right)+{\alpha_{ij}}^{\prime }\left(t\right){d_{ij}}^{\prime }\left(t\right)$$

where ${\vec{g}}_{i,best}^{\prime }$ is the updated global optimal position after chaotic disturbance ∆. The chaotic disturbance rules comply with Equation (12). Thus, both the search step length ${d_{ij}}^{\prime }\left(t\right)$ and search direction ${\alpha_{ij}}^{\prime }\left(t\right)$ are updated. The specific procedure is as follows.

Step #1: This step calculates the fitness value of every seeker in $n_{0}$ iterations and then ranks and obtains the best positions of all the seekers ${\vec{g}}_{i,best}$ in the current population.
Step #2: This step derives *m* noise variables in the neighborhood around ${\vec{g}}_{i,best}$ via chaotic disturbance (${\vec{g}}_{i,best}^{\prime }=\left\{{\vec{g}}_{1,best}^{\prime },\;{\vec{g}}_{2,best}^{\prime },{\vec{g}}_{3,best}^{\prime },\cdots ,\;i\in \left[1,m\right]\right\})$; reappraises the fitness value of ${\vec{g}}_{i,best}^{\prime }$, if ${\vec{g}}_{i,best}^{\prime }<$ ${\vec{g}}_{i,best}$; terminates the disturbance and iterates to the next step; or chooses the best position to replace ${\vec{g}}_{i,best}$.
Step #3: This step updates the search direction via Equations (2) and (4) and updates the search step length via Equations (8) and (9).

**Figure 3:**
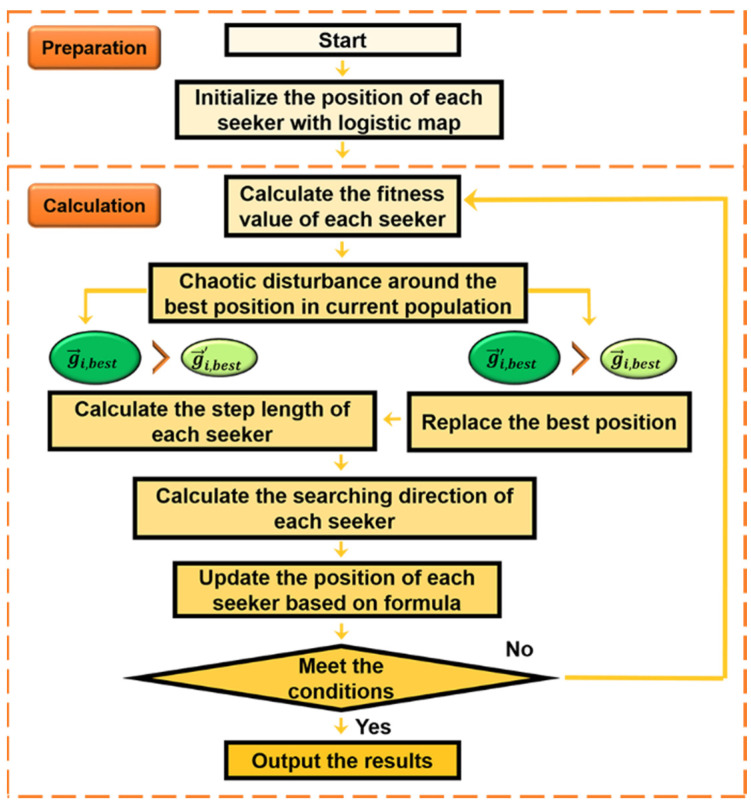
The flowchart of chaotic seeker optimization algorithm.

Moreover, an adaptive inertia weight $w_{1}^{\prime }$ is adopted into the SOA to adjust the step length (in Equation (10)). Unlike [14], the objective function is to obtain the maximum value of the optical energy. Hence, the expression is adjusted as follows:

(17) $$w_{1}^{\prime }=\left\{\begin{array}{c} w_{1max}^{\prime },\;f\leq f_{avg}^{\prime }\;\;\\ w_{1min}^{\prime }+\frac{\left({w_{1max}^{\prime }-w}_{1min}^{\prime }\right)\cdot \left(f_{max}^{\prime }-f\right)}{f_{max}^{\prime }-f_{avg}^{\prime }},\;f\geq f_{avg}^{\prime } \end{array}\right.$$

where $w_{1max}^{\prime }$ and $w_{1min}^{\prime }$ are the maximum and minimum values of $w_{1}^{\prime }$, respectively. $f_{max}^{\prime }$ and $f_{avg}^{\prime }$ are the current population’s maximum and average fitness values, respectively, after chaotic disturbance. The flowchart of the seeker optimization algorithm is updated with the logistic initialization and disturbance support as shown in Figure 3.

## 3. Results and Discussion

The SOA with a linear decreasing inertia weight (LSOA), the DASSS-SOA with a dynamic adaptive step size, and the PSO with a linear decreasing inertia weight (LPSO) were used for the simulation and experiments. Table 1 contains the details of the aforementioned algorithms.

### 3.1. Optical Coupling Model

To evaluate the performance of the CASOA, a simulation and experiments on the coupling alignment between the butterfly optical communication lasers and single-mode fibers were performed. In general, the coupling direction between the lasers and fibers consisted primarily of initial and precise alignments. The laser module was held in place by a clamp, and the fiber was held in place by a six-degree-of-freedom (DOF) movable clamp. During the initial alignment, the fiber moved to incessantly search for the optical signal until the optical power reached certain thresholds. The terminal position was then set as the original position in the precision alignment.

**Figure 4:**
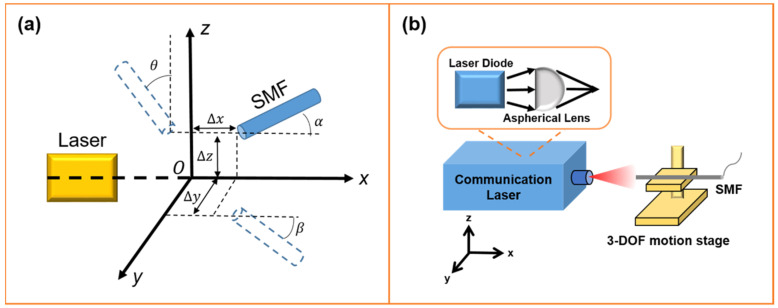
(**a**) The schematic of coupling between lasers and SMFs fixed in 6-DOF motive stage and (**b**) the coupling system of optical communication lasers.

Figure 4a depicts the schematic of laser–SMF coupling in a 6-DOF motive stage, where ∆x,  ∆y, and ∆z are the motion errors of the X, Y, and Z axes, respectively. α, β, and θ represent the alignment angle errors for rotations about the X, Y, and Z axes, respectively.

The schematic of the coupling structure between the optical communication lasers and SMFs is shown in Figure 4b. Laser diodes and aspherical lenses are part of the lasers. Considering that the actual hardware assembly can effectively avoid the coupling loss caused by the angular error, this paper only verified the efficiency and stability of the algorithm in the X, Y, and Z directions in the experiments. Thus, elliptical Gaussian-distributed light emerged from the laser diode, deformed to symmetric Gaussian-distributed light via the aspherical lens, and focused on the fiber end surface. Equation (18) [3,9,23,24] expresses the coupling efficiency model of an optical fiber system.

(18) $$\left\{\begin{array}{c} \eta =\eta_{x}\eta_{y}\\ \eta_{x}=k_{x}exp\left\{-k_{x}\left[\frac{{∆d_{x}}^{2}}{2}\left(\frac{1}{\omega_{x0}^{2}}+\frac{1}{\omega_{f0}^{2}}\right)+\frac{\pi^{2}\theta_{x}^{2}\left(\omega_{x}^{2}\left(z\right)+\omega_{f0}^{2}\right)}{2\lambda^{2}}-\frac{∆d_{x}∆z\theta_{x}}{\omega_{x0}^{2}}\right]\right\}\\ \omega_{x0}^{2}\left(z\right)=w_{x}^{2}\left(1+{\left(\frac{\lambda ∆z}{\pi \omega_{x0}^{2}}\right)}^{2}\right)\\ k_{x}=4\omega_{x0}^{2}\omega_{f0}^{2}/\left[{\left(\omega_{x0}^{2}+\omega_{f0}^{2}\right)}^{2}+\lambda^{2}z^{2}/\pi^{2}\right] \end{array}\right..$$

In Equation (18), η represents the coupling efficiency, which is computed by the overlapping integral of the fields in any plane. $\eta_{x}$ is the coupling efficiency in the vertical junction plane, and $\eta_{y}$ is the coupling efficiency in the parallel junction plane. λ is the wavelength of the laser (1310 nm), $\omega_{f0}$ is the mode field radius of the SMF, and $\omega_{x0}$ is the mode field radius of the LD in the vertical junction plane. $∆d_{x}$ is the linear deviation of the center of the LD and SMF mode field in the vertical junction plane, and ∆z is the motive error for the *Z* axis of the LD and SMF. Because the angular alignment is adjusted by the fixture (shown in Figure 4b), the expression for the coupling efficiency (Equation (18)) is simplified to

(19) $$\left\{\begin{array}{c} \eta =\eta_{1}\cdot \eta_{2}\\ \eta_{1}=\frac{16\omega_{x0}^{2}{\omega_{y0}^{2}\omega }_{f0}^{4}}{\left[{\left(\omega_{x0}^{2}+\omega_{f0}^{2}\right)}^{2}+\lambda^{2}{∆z}^{2}/\pi^{2}\right]\cdot \left[{\left(\omega_{y0}^{2}+\omega_{f0}^{2}\right)}^{2}+\lambda^{2}∆z^{2}/\pi^{2}\right]}\\ \eta_{2}=exp\left\{-k_{x}\left[\frac{{∆x}^{2}}{2}\left(\frac{1}{\omega_{x0}^{2}}+\frac{1}{\omega_{f0}^{2}}\right)\right]-k_{y}\left[\frac{{∆y}^{2}}{2}\left(\frac{1}{\omega_{y0}^{2}}+\frac{1}{\omega_{f0}^{2}}\right)\right]\right\} \end{array}\right..$$

### 3.2. Simulation

This section provides the specific procedure for applying the SOA algorithm to the fiber alignment simulation. The value of η could be used as fitness according to the objective function as shown in Equation (19). Each seeker’s position vector was *x_i_* = (x, y, z), representing the alignment errors in the X, Y, and Z axes. The parameters of the algorithm were then set up for the following calculation (such as the maximum iterative algebra, the total number of seekers, the starting position, and the searching scope). Each seeker’s fitness was calculated and ranked in ascending order. Equations (1)–(5) were used to calculate the direction of each seeker, and Equations (6)–(10) were used to calculate the step length of each seeker. In this case, the ${\underset{d}{\to }}_{pro}$ was set to one in the first iteration. Finally, the position of each seeker was iteratively updated using Equation (11) until the algorithm’s termination condition was met. Table 1 shows the changes in the simulation steps for the CASOA and DASSS-SOA. Table 2 summarizes the parameters that were set in the SOA simulation and IPSO. Then, we tested the search performance of the SOA in the optical search simulation with different numbers of seekers. The maximum iteration *t_max_* was set to 100, and the simulation process was executed 30 times to ensure the algorithm’s robustness.

Based on Equation (19), the optimal value of coupling efficiency was 0.877 when combined with Table 2. Therefore, the terminal condition of algorithm iterations was set to 99% efficiency (0.868). Table 3 shows the search performance of the proposed CASOA with different numbers of seekers.

Table 3 lists the number of successes ($n_{s}$), average iterations ($t_{avg}$), and standard deviations (*std.*) to evaluate the search performance of various algorithms. In detail, $n_{s}$ denotes the search accuracy, $t_{avg}$ denotes the search efficiency, and *std.* denotes the algorithm’s stability. For all the algorithms, as the number of seekers $n_{s}$ increased, $t_{avg}$ and *std.* decreased. When compared with other algorithms, the proposed CASOA algorithm performed well in terms of the search accuracy and efficiency with small seekers (particles). The CASOA successfully achieved the terminal coupling efficiency of 26 times with two seekers (86.7%). However, the DASSS-SOA only achieved 60%, the LSOA only achieved 43.3%, and the PSO failed nearly all the tests. When there were more than 10 seekers, the information exchange between the seekers in the PSO algorithm became very useful, and the success rate increased. The results of $n_{s}$ show that the SOA had better search area properties in a single particle than the PSO algorithm. Among the SOAs, the CASOA generated multiple seekers through chaotic disturbance to increase communication in each iteration and jumped to the local optimum. The DASSS-SOA adaptively adjusted the step length (expression in Table 1) to balance the global and local search properties. However, the adjustment performance was inferior to that of the CASOA, particularly in the case of a small number of seekers. When more than 20 seekers were used to search for the optical signal, the LPSO outperformed the other SOAs. The $t_{avg}$ and *std.* of the LPSO were clearly lower than those of the SOAs, especially when the number of seekers exceeded 40. In comparison to the SOAs, the results show that the LPSO relied more on the population.

**Figure 5:**
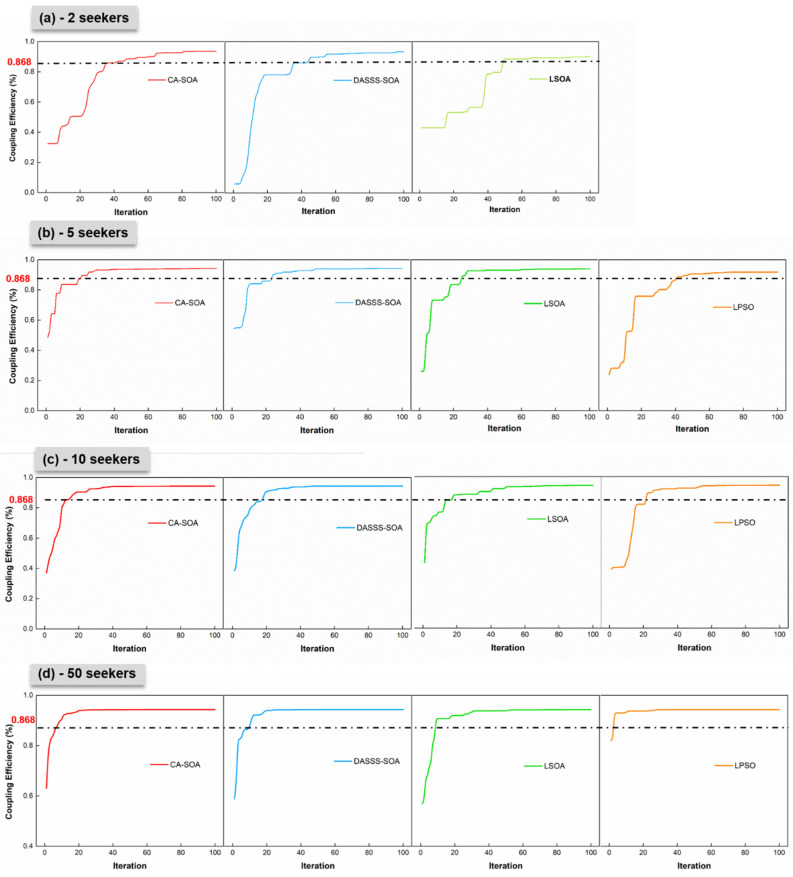
Comparison of different algorithms with a population in the optical coupling simulation: (**a**) 2 seekers, (**b**) 5 seekers, (**c**) 10 seekers, and (**d**) 50 seekers.

In general, the curves on the relationship between the algorithm iteration and fitness value show that the algorithms dropped into local optimums and jumped out during the search process. When the fitness value remained constant as the number of iterations increased, the algorithm reached a local optimum. Figure 5 depicts the search process of various algorithms in the simulation with average iterations. According to Figure 5a,b, when only a few seekers searched for the fitness value, the algorithm fell into a local optimum before reaching the terminal condition (η = 0.868). The CASOA could quickly adjust to chaotic disturbances and escape from the local optimum. Meanwhile, the other algorithms required several iterations to escape from the local optimum. It could be attributed to the decrease in the coupling efficiency. With the promotion of seekers, the search curves became smoother, and the step shape gradually disappeared in Figure 5c,d. The acceleration of the population, particularly for the LPSO algorithm, significantly improved the search performance.

When combined with Table 3 and Figure 5, the simulated results show that population acceleration could avoid falling into a local optimum. However, many seekers necessitated additional calculations of the fitness value in each iteration. In the LPSO, for example, 50 particles (seekers) required only 2.9 iterations to reach the target. Consequently, the number of detected spatial points was calculated to be 145. In total, 10 seekers in the CA-SOA required 15.5 iterations to achieve the target. Thus, the number of detected spatial points (155) was close to that of the LPSO. The proposed CASOA, which had a good search performance in a small population, could provide another strategy for increasing the coupling efficiency. Because the simulation did not account for the length of the search path, the final result had to be verified experimentally.

### 3.3. Experiment

**Figure 6:**
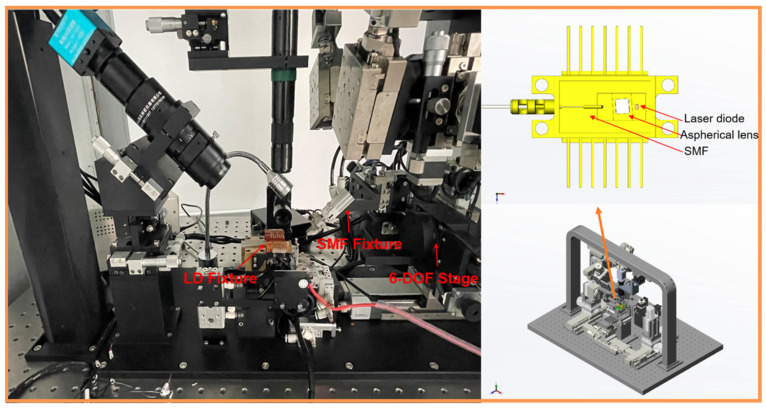
Three-dimensional (3D) graphic views of the butterfly communication laser coupling and packaging equipment.

The experimental system comprised a comprehensive set of butterfly coupling and packaging equipment. The specific components included a 1310 nm semiconductor laser as the input source, a 6-DOF motion platform for coupling and alignment, two fixtures to accommodate an LD and an SMF, an optical power meter for testing the optical output power, a laser diode and a single-mode fiber for experiment preparation, and other communication modules. Figure 6 depicts the butterfly communication laser’s sectional equipment and structures. The optical power meter measured the highest laser output power in the range of 750 μW–1000 μW. Thus, the terminal condition threshold was set to 700 μW.

The specific experimental process was as follows:

Step #1: With CCD recognition, the SMF was shifted to a position where the optical output power could reach up to 10 μW. This position was then designated as the initial coupling point.
Step #2: The platform’s repeatability was set to 0.1 μm, and the movement velocity was set to 1 mm/s. The search range was set to three dimensions: $x\in \left(-10\;\mu m,10\;\mu m\right),\;y\in \left(-10\;\mu m,10\;\mu m\right),\;and\;z\in \left(-25\;\mu m,25\;\mu m\right)$. Thus, this search space could generate 20,000 unshrouded points. The precision alignment algorithms were then applied to the movement system. The fitness value was defined as the optical power value, and the search could last up to 700 μW or 150 iterations. The number of seekers was set between 10 and 30, and the other parameters of the algorithms used in this experiment were the same as in the simulation (Table 2). In the CASOA, for example, we set 10 seekers as the chaotic disturbance population, and the chaotic map was limited to 1μm×1μm×1μm.
Step #3: The 3-DOF movement platform moved continuously when the coupling experiment started. The SMF moved to the target points stepwise in each iteration based on the algorithms. Then, the SMF shifted to the largest optical output power of the searched point for the next iteration after comparing all the optical output powers of the searched point.
Step #4: The SMF finally arrived at the final target point. It recorded all the largest points in each iteration and the corresponding search time.

**Figure 7:**
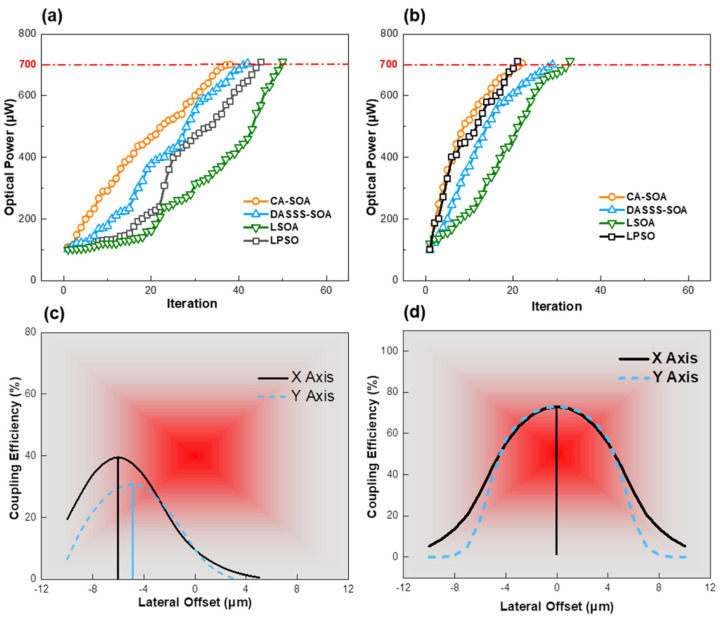
Experimental results of laser diode (LD) and SMF optical power search with several algorithms (**a**) with 10 seekers and (**b**) with 30 seekers; (**c**) the output optical power distribution of X and Y axes in initial alignment; (**d**) the output optical power distribution of X and Y axes after precise alignment by chaotic adaptive seeker optimization algorithm (CASOA).

Each algorithm was employed in the coupling experiment 10 times, and the average iterations and time were collected. The experimental results are clearly shown in Table 4 and Figure 7.

Table 4 shows that the experimental average iterations $\left(t_{avg}^{\prime }\right)$ are clearly greater than those of the simulated results. Furthermore, the search accuracy (or success rate) in the experiments was lower than that in the simulations. One possible attribution was the motion error. The superposition of errors occurred due to motion errors in a 3-DOF movement platform. Consequently, the detected point in the previous iteration may have caused the deviation in the subsequent iteration. The experimental results in Table 4 show that the CASOA had the best performance among the algorithms in terms of search accuracy and efficiency. It took 38.3 iterations (10 seekers) and 23.4 iterations (30 seekers) to reach the optical power threshold with a 100% success rate. Meanwhile, the LPSO and DASSS-SOA only had good search performances with 30 seekers and with 22.8 and 29.4 average iterations, respectively. The LSOA required the most iterations to reach the target value and had the lowest efficiency and accuracy with 10 and 30 seekers. The search time was the most surprising aspect of the results. The CASOA completed the search experiment after 32.3 iterations with a search time of 12.8 s and with a population of 10 seekers. Meanwhile, the LPSO used 22.8 iterations to achieve the target in 13.6 s with a population of 30 seekers. This implies that it takes longer to complete fewer iterations. This finding contradicts previous studies [9,25], which reported that optimal iterations are the most important criteria in coupling efficiency. One possible explanation for this is that the seekers in the CASOA had less displacement than those in the LPSO due to iteration augmentation. Furthermore, the number of detected spatial points in the CASOA was lower than that in the PLSO. Meanwhile, the detected spatial points were associated with 3-DOF displacement. More displacement took more time when moving at the same velocity. Consequently, the displacement of 10 seekers in the CASOA was less than that of 30 seekers in the LPSO.

Figure 7a,b show a comparison of the above algorithms in the coupling of the LD and SMF. According to the data in Figure 7, the attribution of iteration reduction was population growth. All the algorithms with a sufficient population had good search performance in the early iterative stage. With a small population, the DASSS-SOA, LSOA, and LPSO were likely to be trapped into a local optimum, impeding the continuous optical power search. The CASOA’s search curves were smooth and nonstep, indicating that the chaotic map and disturbance assisted the CASOA in adjusting the balance of the global and local search. The CASOA could quickly obtain information about the best position with the most optical power, especially in the early stages. In general, an early search performance affected the results, which was more noticeable in small populations. The relationship between the optical power and lateral offset is depicted in Figure 7c,d. The X and Y axes deviated 6 μm and 4 μm from the global best position in the initial stage (after initial alignment). The coupling efficiency was only 40%. The X and Y axes moved to the best positions after the CASOA induced precise alignment. The coupling efficiency was 74%. The experimental results show that the CASOA had excellent performance (in terms of accuracy and efficiency) in the precise search of LD and SMF coupling, with small and large populations. Similarly, the DASSS-SOA also performed well with a large population. However, it always became trapped in a local extreme in the early stages when the population was small. The LPSO and LSOA algorithms performed poorly in the early stages with a small population size. The LPSO used powerful information exchange to improve the search performance when the population size was large, but the LSOA failed. These findings were consistent with the simulation.

Overall, the simulated and experimental results show that the CASOA performed well in the LD and SMF coupling alignment. The coupling efficiency was significantly increased by the powerful search property with a small population. In the iteration process, we introduced some chaotic disturbances to ensure that the best position had the highest fitness value compared with linear decreasing laws (the LPSO and LSOA). Consequently, when seekers became trapped in a local extreme, they could escape on their own [26,27]. Lin et al. (2014) [26] created an opposite space to replicate the search position in each iteration. It computes the fitness value of two spaces and updates the better position. Even though this method improves the global search property, the detected spatial points reduce the search efficiency. The proposed CASOA constrained the disturbance space around ${\vec{g}}_{i,best}$. Thus, it could assist seekers in efficiently escaping the local optimum. The LPSO could also increase the population size to reduce the iteration time. Therefore, it performed well in terms of search accuracy and efficiency in a 30-seeker experiment (Figure 7b). However, the universality of search in the early stages resulted in longer displacement and more motion errors. The basis of the global search in several swarm optimization studies is a mass of particles. We proposed this method to maintain the global search property with small particles. Finally, the proposed CASOA can be used in LD and SMF coupling alignments. The excellent coupling efficiency and accuracy are beneficial for optical device packaging. In the meantime, we believe that this method can provide theoretical support in swarm optimization problems.

## 4. Conclusions

The chaotic map and chaotic disturbance were successfully integrated with the adaptive seeker optimization algorithm. The coupling accuracy and efficiency of a butterfly optical communication laser were improved with the help of CASOA optimization. A series of simulated and experimental results validated the algorithm’s optimizing performance. Furthermore, the search strategies with large and small populations were compared and discussed. Finally, the following conclusions were drawn.

(1) The CASOA exhibited a good optimization performance with a small population (accuracy and efficiency). It only took 38.3 iterations and 14.7 s to arrive at the best position with the highest output optical power and a search accuracy of 100%. The DASSS-SOA, LSOA, and LPSO performed poorly compared with the CASOA. The chaotic disturbance had the potential to enhance the optimizability of seekers, particularly in the early stages.
(2) Increasing the population to decrease the number of iterations could yield good results for LD and SMF coupling alignment. However, the number of detected points increased, resulting in more displacement and motion errors. Consequently, few population strategies in the CASOA could reduce displacement and, consequently, motion errors. This method also improved the coupling efficiency.

## Figures and Tables

**Table 1:** The introduction of algorithms for the comparison of optimal performance.

Algorithm	Function Variation	Parameters	Reference
DASSS-SOA	$w_{1}=\left\{\begin{array}{c} w_{1max},\;f\leq f_{avg}\;\;\\ w_{1min}+\frac{\left(w_{1max}-w_{1min}\right)\cdot \left(f_{max}-f\right)}{f_{max}-f_{avg}},\;f\geq f_{avg} \end{array}\right.$	$w_{1max}=0.9$ $w_{1min}=0.1$	Yang H., 2020 [14]
LSOA	$w_{1}=\left(t_{max}-t\right)/t_{max}$	$t_{max}=100$	Dai C.H., 2010 [10]
LPSO	$v_{id}^{k}=w\cdot v_{id}^{k-1}+c_{1}\cdot r_{1}\cdot \left(p_{id}-x_{id}^{k-1}\right)+c_{2}\cdot r_{2}$ $\cdot \left(p_{gb}-{\;\;x}_{id}^{k-1}\right)$ $w_{1}=w_{max}-\left(w_{max}-w_{min}\right){\left(y_{b}/y_{f}\right)}^{\alpha }$	$w_{max}=0.9$ $w_{min}=0.4$ α=2 $c_{1}=c_{2}=2$	Lian D., 2021 [9]

**Table 2:** Simulated parameters set in the seeker optimization algorithm (SOA) and particle swarm optimization (PSO) algorithm.

SOA				Improved PSO			
Parameter	Value	Parameter	Value	Parameter	Value	Parameter	Value
$\omega_{x0}$	3 μm	$\omega_{max}$	0.9	$\omega_{x0}$	3 μm	$\omega_{min}$	0.4
$\omega_{y0}$	5 μm	$\omega_{min}$	0.1	$\omega_{y0}$	5 μm	*x*	$\left(-10,10\right)$
$\omega_{f0}$	4 μm	*x*	$\left(-6,6\right)$	$\omega_{f0}$	4 μm	*y*	$\left(-10,10\right)$
λ	1.31 μm	*y*	$\left(-6,6\right)$	λ	1.31 μm	*z*	$\left(-300,300\right)$
$u_{max}$	0.9500	*z*	$\left(-300,300\right)$	$c_{1}$	2	$\theta_{x}$	$\left(-7{}^{\circ},7{}^{\circ}\right)$
$u_{min}$	0.0111	$\theta_{x}$	$\left(-7{}^{\circ},7{}^{\circ}\right)$	$c_{2}$	2	$\theta_{y}$	$\left(-7{}^{\circ},7{}^{\circ}\right)$
μ	4	$\theta_{y}$	$\left(-7{}^{\circ},7{}^{\circ}\right)$	$\omega_{max}$	0.9	\	\

**Table 3:** The simulated results using the SOA and PSO algorithm with different numbers of seekers.

CASOA in Simulation Performed 30 Times								
$\mathrm{Number}\;\mathrm{of}\;\mathrm{seekers}\;\left(p\right)$	2	5	10	20	30	40	50	100
$\mathrm{Number}\;\mathrm{of}\;\mathrm{successes}\;\left(n_{s}\right)$	26	30	30	30	30	30	30	30
$\mathrm{Average}\;\mathrm{iterations}\;\left(t_{avg}\right)$	42.3	19.2	15.5	12.3	9.8	8.4	7.2	4.6
$\mathrm{Standard}\;\mathrm{deviations}\;\mathrm{of}\;\mathrm{iterations}\;\left(std.\right)$	16.986	9.460	6.274	2.938	2.878	2.923	2.679	2.813
DASSS-SOA in simulation performed 30 times								
$\mathrm{Number}\;\mathrm{of}\;\mathrm{seekers}\;\left(p\right)$	2	5	10	20	30	40	50	100
$\mathrm{Number}\;\mathrm{of}\;\mathrm{successes}\;\left(n_{s}\right)$	18	30	30	30	30	30	30	30
$\mathrm{Average}\;\mathrm{iterations}\;\left(t_{avg}\right)$	44.0	23.9	18.8	14.1	13.9	12.6	10.2	7.1
$\mathrm{Standard}\;\mathrm{deviations}\;\mathrm{of}\;\mathrm{iterations}\;\left(std.\right)$	16.213	9.179	8.612	4.813	4.323	4.014	3.145	3.328
LSOA in simulation performed 30 times								
$\mathrm{Number}\;\mathrm{of}\;\mathrm{seekers}\;\left(p\right)$	2	5	10	20	30	40	50	100
$\mathrm{Number}\;\mathrm{of}\;\mathrm{successes}\;\left(n_{s}\right)$	13	30	30	30	30	30	30	30
$\mathrm{Average}\;\mathrm{iterations}\;\left(t_{avg}\right)$	49.8	23.4	17.2	12.1	13.9	12.6	8.5	5.6
$\mathrm{Standard}\;\mathrm{deviations}\;\mathrm{of}\;\mathrm{iterations}\;\left(std.\right)$	19.804	11.093	8.457	4.873	3.786	3.823	3.145	2.715
LPSO in simulation performed 30 times								
$\mathrm{Number}\;\mathrm{of}\;\mathrm{seekers}\;\left(p\right)$	2	5	10	20	30	40	50	100
$\mathrm{Number}\;\mathrm{of}\;\mathrm{successes}\;\left(n_{s}\right)$	1	25	30	30	30	30	30	30
$\mathrm{Average}\;\mathrm{iterations}\;\left(t_{avg}\right)$	46	43.3	23.3	9.8	5.8	3.6	2.9	1.8
$\mathrm{Standard}\;\mathrm{deviations}\;\mathrm{of}\;\mathrm{iterations}\;\left(std.\right)$	-	22.892	11.089	6.660	5.323	4.325	1.912	0.554

**Table 4:** Coupling experimental results using different algorithms.

10 Seekers	CASOA	DASSS-SOA	LSOA	LPSO
Number of successes	10	9	7	8
Number of failures	0	1	3	2
$\mathrm{Average}\;\mathrm{iterations}\;\left(t_{avg}^{\prime }\right)$	38.3	42.6	50.4	48.2
The number of detected spatial points	383	426	504	482
Search time (s)	14.7	16	20.2	19.1
30 seekers	CASOA	DASSS-SOA	LSOA	LPSO
Number of successes	10	10	9	10
Number of failures	0	0	1	0
$\mathrm{Average}\;\mathrm{iterations}\;\left(t_{avg}^{\prime }\right)$	23.4	29.4	33.6	22.8
The number of detected spatial points	702	882	1008	684
Search time (s)	14.6	16.2	17.2	13.6
